# High-Precision Demodulation Method for Multi-FP Cavity Sensors Based on Filter Optimization

**DOI:** 10.3390/s26185688

**Published:** 2026-09-08

**Authors:** Tianchi Qiao, Qing Shi, Buqiang Zhang, Cong Ma, Guopei Mao

**Affiliations:** Beijing Research Institute of Telemetry, Beijing 100076, China; qtianchi@163.com (T.Q.); zhangbq@brit.com.cn (B.Z.); macong0520@126.com (C.M.); maoguopei@163.com (G.M.)

**Keywords:** multi-FP cavity demodulation, spectral separation, high-precision demodulation, filtering

## Abstract

Fibre-optic Fabry–Pérot (FP) multi-cavity sensors hold significant promise for multi-parameter measurements in extreme environments. However, precisely demodulating signals from each cavity within the composite interference spectrum remains a key bottleneck that limits measurement accuracy. To address this issue, this paper proposes a high-precision demodulation method that combines digital filtering with distortion region suppression. This method first uses digital bandpass filters to separate the interference signals from each sub-cavity from the composite spectrum. It then removes the spectral regions distorted by the filters, retaining only the stable central spectral band for interference order fitting and cavity length calculation. To comprehensively evaluate the method, we systematically compared the distortion characteristics of six finite impulse response (FIR) window-function filters and three infinite impulse response (IIR) filters during spectral separation, along with their impact on demodulation accuracy. Simulation results show that, within the 490–510 μm cavity-length range, FIR filtering combined with distortion suppression reduces the demodulation error to below 0.04 nm, approaching the theoretical limit. In contrast, the best-performing IIR filter still yields an error of 0.58 nm after the same processing. Furthermore, in simulations covering a wide range of 200–800 μm, the maximum demodulation error of this method is less than 0.53 nm, demonstrating its excellent robustness. In dual-cavity temperature sensing experiments, the partial-spectrum demodulation strategy with distortion suppression reduced the standard deviation of cavity-length fluctuations from 0.112 nm to 0.072 nm, and the maximum adjacent point jump from 2.158 nm to 0.464 nm, compared with full-spectrum demodulation. This significantly enhances the continuity and stability of demodulation. This study not only provides a high-precision, highly robust demodulation scheme for multi-cavity FP sensors but also offers clear theoretical and experimental grounds for selecting and optimising digital filters in practical engineering applications.

## 1. Introduction

Fiber-optic Fabry-Pérot (FP) sensors have become one of the most important sensing technologies because of their compact size, simple fabrication, and excellent immunity to electromagnetic interference. These sensors can operate reliably in harsh environments, such as high-temperature and high-pressure conditions, and can measure multiple physical parameters. Consequently, FP sensors have been widely used to measure temperature [1], strain [2], pressure [3], and refractive index [4], and have found broad applications in defence [5], aerospace [6], deep-sea exploration [7], and biomedicine [8].

With the increasing demand for multi-parameter sensing in complex environments, conventional single-parameter FP sensors are no longer sufficient for many practical applications. In particular, under harsh conditions such as high temperatures and high pressures, cross-sensitivity among different physical parameters can significantly degrade measurement accuracy and system reliability. To overcome this limitation, researchers have developed various multi-FP cavity sensor configurations for simultaneous multi-parameter measurement. Various demodulation techniques have been developed specifically for multi-cavity FP sensors. They can be broadly classified into three groups. The first group employs frequency-domain analysis, typically using the fast Fourier transform (FFT) to separate the frequency components of different cavities, and then calculates the cavity length from the peak positions or phase information in the Fourier domain [9]. The second group uses digital filtering to extract individual cavity spectra from the composite interference spectrum, and then applies conventional single-cavity demodulation algorithms [10,11]. The third group comprises phase-demodulation techniques based on low-coherence interferometry or multi-wavelength interrogation. These techniques enable high-speed dynamic measurements by directly tracking phase changes [12,13,14]. Recent studies have made notable progress in addressing the multi-cavity demodulation challenge from different perspectives. Deng et al. [12] proposed a frequency-shift technique for dual-cavity FP sensors: by shifting the frequency of the combined cavity’s reflection spectrum toward a lower frequency direction, they obtain a stronger reflection spectrum of the short FP cavity, effectively improving the signal-to-noise ratio and demodulation accuracy of the weak short-cavity component. Ren et al. [13] developed a three-wavelength demodulation technique specifically for multi-cavity FP sensors. This technique uses broadband optical filters to ensure that interference occurs only in the shortest cavity, based on the principle of low-coherence interference, and enables high-speed dynamic signal extraction at 500 kHz. Li et al. [14] demonstrated a real-time pressure measurement method for multi-cavity FP sensors. The method uses birefringent crystals and polarizers to obtain orthogonal signals, and applies a four-quadrant inverse tangent operation for rapid phase demodulation at 5 kHz, which significantly reduces the influence of irrelevant cavities on the demodulation results. While these methods have advanced multi-cavity FP demodulation—offering improvements in SNR, dynamic speed, or real-time performance—they rely on the premise that the interference spectra of individual cavities can be reliably extracted.

FFT-based digital filtering has been widely adopted for spectral separation in multi-FP cavity demodulation. However, most existing studies primarily focus on signal separation and overlook the spectral distortion introduced by the filtering process itself. Such distortion—including edge distortion and peak shifts—can significantly degrade the accuracy of subsequent cavity-length demodulation, yet this issue has received little attention in existing multi-cavity demodulation studies. Therefore, understanding the distortion characteristics of different filters and developing appropriate compensation strategies is essential for achieving high-precision demodulation.

To address this gap, this paper systematically investigates the distortion mechanisms introduced by FIR and IIR filters through a comparative analysis of six FIR window functions and three IIR filters. We reveal that FIR filtering predominantly introduces local boundary distortion, whereas IIR filtering causes global spectral distortion. Based on this finding, we propose a distortion-rejection strategy that discards the distorted boundary peaks and retains only the stable central spectral band for interference-order fitting. Comprehensive simulations and dual-cavity temperature experiments are performed to evaluate the proposed strategy. The results demonstrate that it effectively suppresses FIR-induced errors but provides only limited improvement for IIR filters. These findings not only provide an effective method for high-precision demodulation of multi-FP cavity sensors but also offer quantitative guidance for selecting and optimising digital filters in practical engineering applications.

## 2. Theory

**Figure 1:**
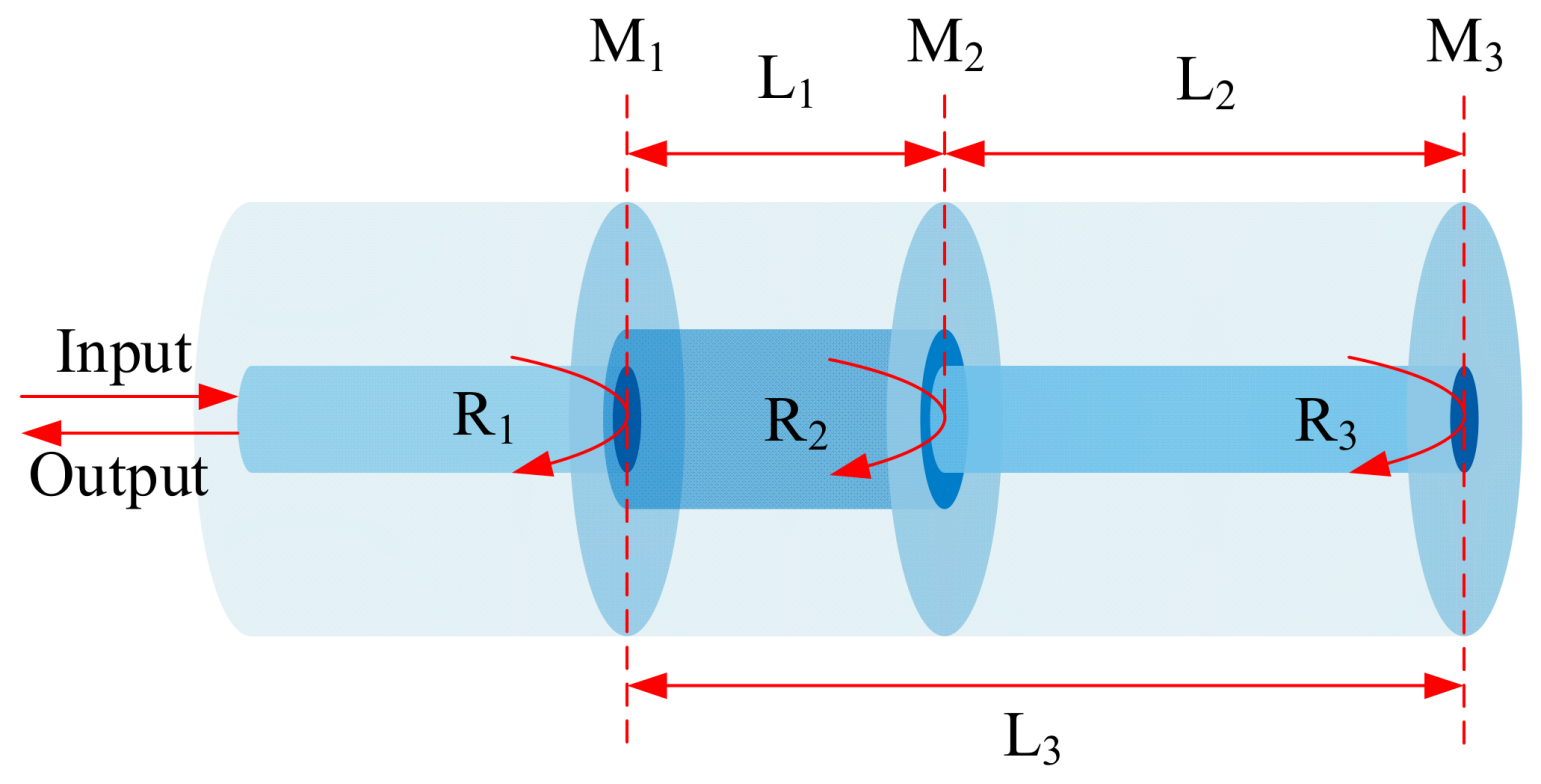
Schematic diagram of a composite FP cavity sensor structure.

The composite FP cavity sensor consists of three parallel reflective surfaces, as illustrated in Figure 1.

Reflective surfaces *M*_1_ and *M*_2_ form the first FP cavity (*FPI*_1_) with a cavity length of *L*_1_, while *M*_2_ and *M*_3_ form the second FP cavity (*FPI*_2_) with a cavity length of *L*_2_. The third FP cavity is formed by *M*_1_ and *M*_3_ with a cavity length of *L*_3_. The resulting interference intensity can be expressed as:

(1) $$\begin{array}{c} I=R_{1}+R_{2}+R_{3}+2\sqrt{R_{1}R_{2}}\cos\left(\frac{4\pi n_{1}L_{1}}{\lambda }\right)\\ \;+2\sqrt{R_{2}R_{3}}\cos\left(\frac{4\pi n_{2}L_{2}}{\lambda }\right)\\ \;+2\sqrt{R_{1}R_{3}}\cos\left(\frac{4\pi \left(n_{1}L_{1}+n_{2}L_{2}\right)}{\lambda }\right) \end{array}$$

where *R*_1_, *R*_2_, and *R*_3_ is the reflectivities of surfaces *M*_1_, *M*_2_, and *M*_3_, *n*_1_ and *n*_2_ are the refractive indices of *FPI*_1_ and *FPI*_2_, and *λ* is the wavelength.

Spectral filtering is first performed to separate the interference spectra of individual FP cavities from the composite spectrum. The frequency response of an ideal bandpass filter is given by (2), where ω_0_ and ω_1_ are the lower and upper cutoff frequencies. The passband gain is unity, whereas the stopband gain is zero.

(2) $$H\left(\omega \right)=\left\{\begin{array}{l} 1,\omega_{0}\leq \left|\omega \right|\leq \omega_{1}\\ 0,else \end{array}\right.$$

Because digital filtering inevitably introduces distortion near the spectral boundaries, only the stable central portion of the filtered interference spectrum is retained for subsequent interference-order fitting. This distortion-rejection step effectively suppresses the demodulation errors caused by local spectral distortion while preserving the intrinsic interference characteristics.

After the interference spectrum of an individual FP cavity has been extracted, the cavity length is estimated using the interference-order fitting method. The interference spectrum of a single FP cavity can be expressed as:

(3) $$I=R_{1}+R_{2}+2\sqrt{R_{1}R_{2}}\cos\left(\frac{4\pi nL}{\lambda }\right)$$

**Figure 2:**
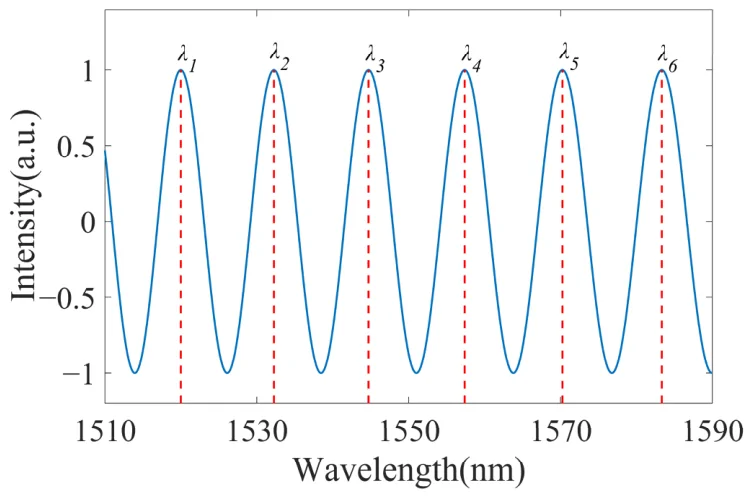
Single FP cavity interference spectrum.

The simulated interference spectrum is shown in Figure 2.

Assuming that the wavelengths corresponding to successive interference peaks are *λ*_1_,*λ*_2_,…,*λ_N_*, the following relationship is obtained:

(4) $$\frac{2nL}{\lambda }=m$$

where m is a positive integer denoting the interference order. By introducing the wavenumber *v_N_* = 1/*λ_N_*, (4) can be rewritten as:

(5) $$2nL\nu_{N}=m$$

Accordingly,

(6) $$\left\{\begin{array}{l} 2nL\nu_{N}\;\;\;=m\;\\ 2nL\nu_{N-1}=m+1\\ \ldots \ldots \\ 2nL\nu_{1}\;\;\;=m+\left(N-1\right)\; \end{array}\right.$$

Dividing both sides of (6) by 2*L* yields:

(7) $$\left\{\begin{array}{l} \nu_{N}\;\;\;\;=m\times \frac{1}{2nL}\;\\ \nu_{N-1}=m\times \frac{1}{2nL}+1\times \frac{1}{2nL}\\ \ldots \ldots \\ \nu_{1}\;\;\;\;=m\times \frac{1}{2nL}+\left(N-1\right)\times \frac{1}{2nL} \end{array}\right.$$

Equation (7) can be rewritten in the linear form *y* = *ax* + *b*, where *y* = {*v_N_*,*v_N_*_−1_,…,*v*_1_}, *x* = {0,1,…,N−1}, and the coefficients a and b are given by *a* = 1/2*nL*, *b* = *m*/2*nL*. The coefficients are estimated using linear least-squares fitting, from which the interference order is determined.

(8) $$\tilde{m}=\frac{b}{a}$$

Because the interference order estimated from the measured spectrum is generally non-integer, it is rounded to the nearest integer to obtain *m*.

(9) $$m=\left[\tilde{m}\right]$$

Based on (7), the expression for *L* can be derived as:

(10) $$\left\{\begin{array}{l} L=m\times \frac{1}{2n\nu_{N}}\\ L=\left(m+1\right)\times \frac{1}{2n\nu_{N-1}}\\ \ldots \ldots \\ L=\left(m+N-1\right)\times \frac{1}{2n\nu_{1}} \end{array}\right.$$

Finally, the cavity length is calculated by averaging the values obtained from all interference peaks.

(11) $$L=\overset{N-1}{\underset{i=0}{\sum }}\left(m+i\right)\times \frac{1}{2n\nu_{N-i}}$$

In interferometric order-fitting demodulation algorithms, high accuracy depends on accurately determining the wavenumbers of each peak in the interferometric spectrum. Theoretically, if the sampling density is sufficiently high, the peak positions extracted directly from the discrete sampling points can approximate the true values and thus achieve high demodulation accuracy. However, increasing the number of sampling points significantly increases the volume of spectral data and reduces algorithmic efficiency.

**Figure 3:**
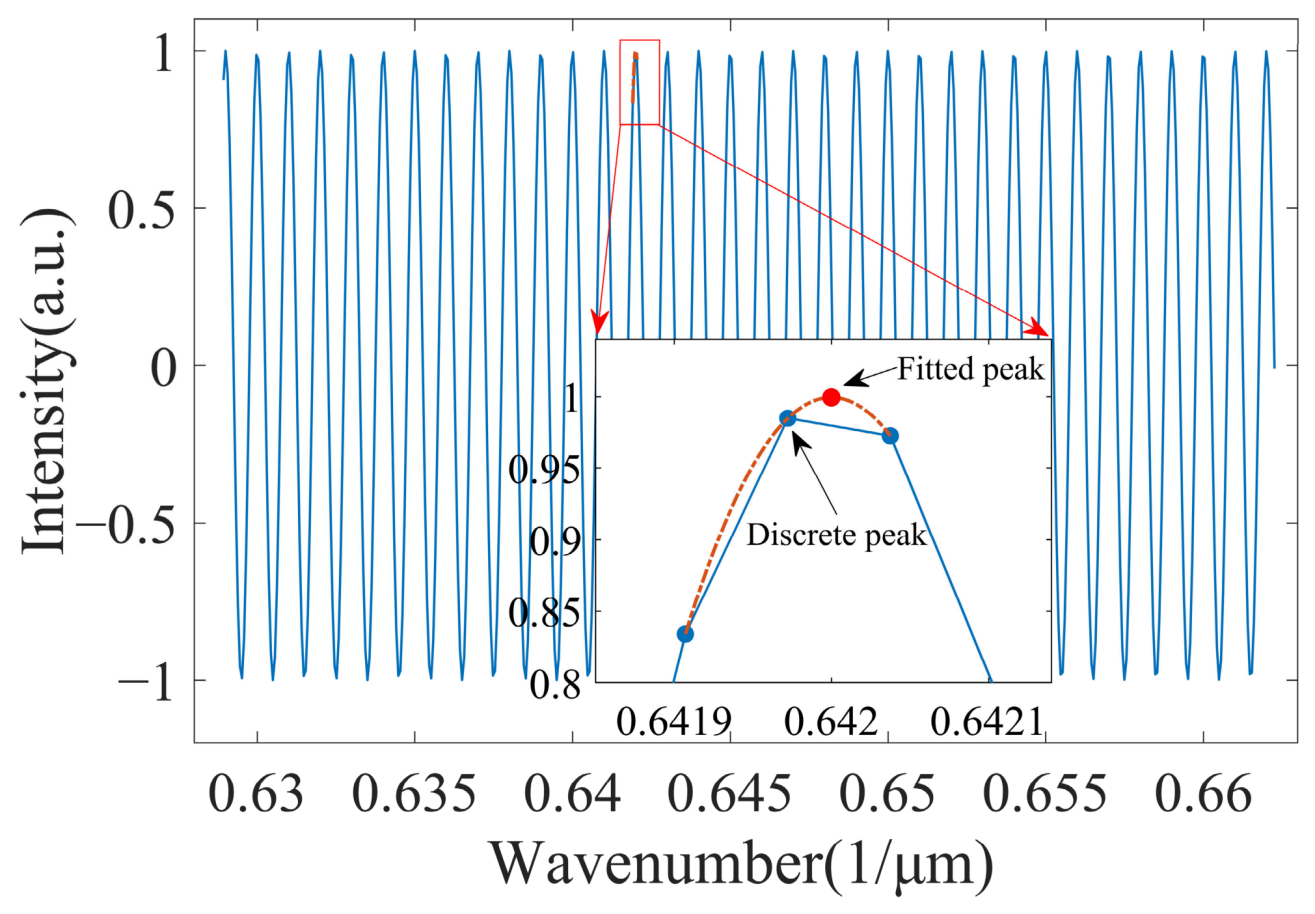
Schematic diagram of peak localisation based on a quadratic polynomial fit using three data points.

To address this issue, this paper uses a peak localisation method based on a three-point quadratic polynomial fit, as shown in Figure 3. First, a peak-finding algorithm locates discrete peak points. Then, one point to the left and one to the right of each peak point—three discrete points in total—are selected for a quadratic fit. The vertex of the resulting parabola is taken as the new peak position.

## 3. Simulations

**Figure 4:**
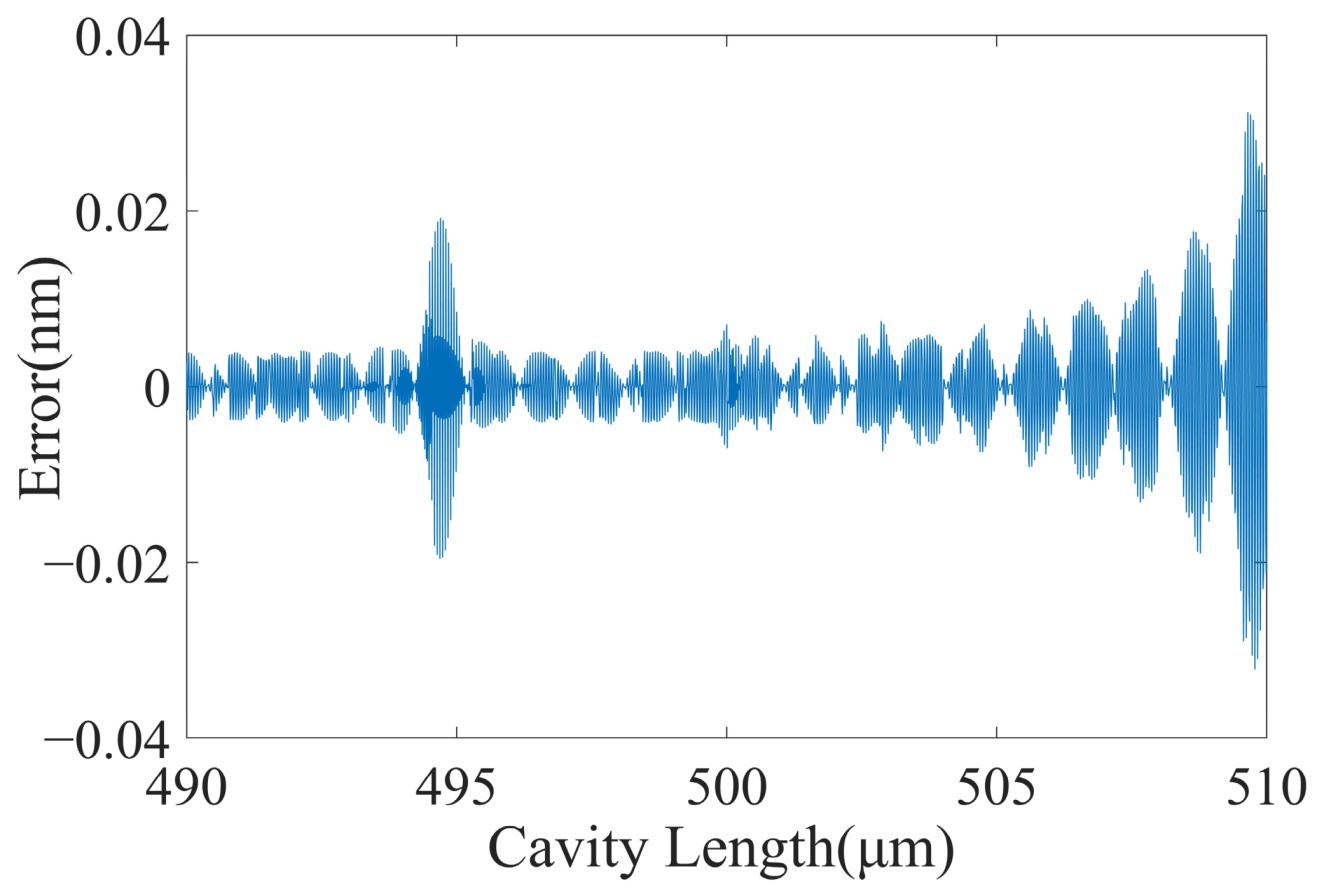
Demodulation accuracy of the algorithm.

The proposed demodulation algorithm was first evaluated using a simulated interference spectrum. The simulated spectrum covered a wavelength range of 1510 to 1590 nm and contained 512 sampling points. To evaluate the accuracy of the algorithm, no noise was added during the simulation. We created interferograms for FP cavity lengths ranging from 490 μm to 510 μm, in steps of 1 nm. Demodulation was performed using an interference-order fitting algorithm. The results are shown in Figure 4.

Under ideal conditions, the proposed algorithm achieved an average demodulation error of −1.118 × 10^−3^ nm, a standard deviation (SD) of 0.0054 nm, and a maximum error of 0.032 nm, corresponding to an overall demodulation accuracy of better than 0.04 nm.

**Figure 5:**
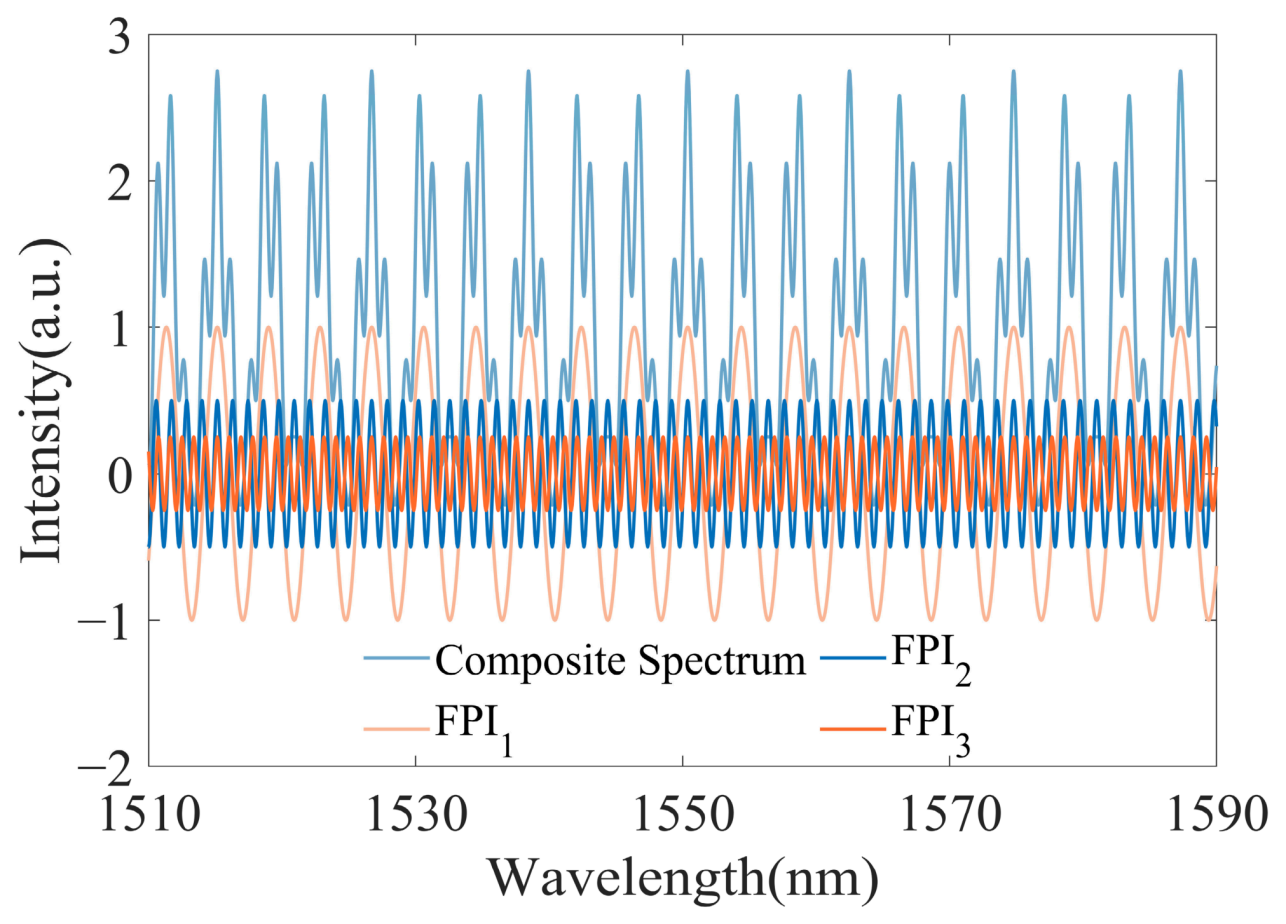
Simulated interference spectrum.

To evaluate the spectral separation performance of the proposed filters, a composite interference spectrum was generated according to the model in (1). The simulated spectra are shown in Figure 5.

Figure 5 presents the individual interference spectra of *FPI*_1_, *FPI*_2_, and *FPI*_3_, together with the composite spectrum obtained by superimposing the three components. The cavity length of *FPI*_1_ was varied from 490 to 510 μm, while *FPI*_2_ was fixed at 1000 μm. *FPI*_3_ corresponds to the cavity formed between the first and third reflective surfaces.

**Figure 6:**
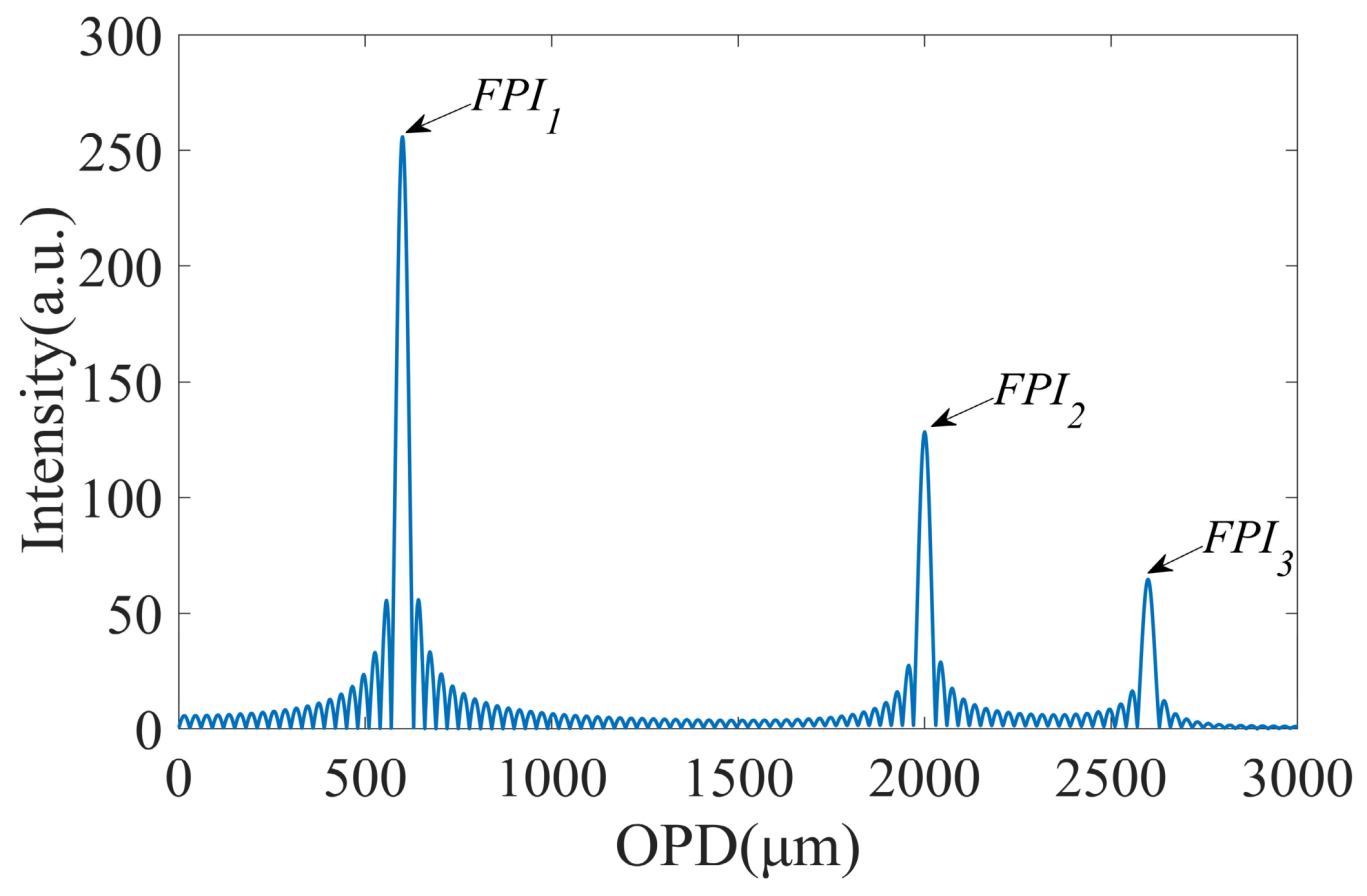
FFT of the composite cavity spectrum.

The composite spectrum contains multiple overlapping frequency components, which makes direct cavity-length demodulation impractical. Subsequently, the composite spectrum is converted from the wavelength domain to the wavenumber domain (*v* = 1/*λ*), and a fast Fourier transform (FFT) is performed on it. The schematic diagram of the amplitude spectrum is shown in Figure 6.

The three distinct peaks in the frequency spectrum correspond to *FPI*_1_, *FPI*_2_, and *FPI*_3_, respectively. By applying appropriately designed band-pass filters, the interference spectrum of each individual cavity can be reconstructed, thereby separating the composite multi-cavity signal. To accomplish this, both FIR and IIR band-pass filters were designed and evaluated. According to the FFT spectrum, the frequency component of *FPI*_1_ was identified and subsequently used as the center frequency for band-pass filter design. The specific filter parameters are shown in Table 1.

For the FIR filters, six window functions were employed: Hann, Hamming, Blackman, Blackman–Harris, Bartlett, and Gaussian. The filter order for each window function was set to 36, 30, 40, 46, 32, and 32, respectively, with the corresponding number of taps equal to the order plus one. The Gaussian window was implemented with α = 2.5 (the default value). For the IIR filters, three types were designed: Butterworth, Chebyshev Type I, and Chebyshev Type II. All three IIR filters are of first order. The passband ripple (Rp) of the Chebyshev Type I filter is set to 0.1 dB, while the stopband attenuation (Rs) of the Chebyshev Type II filter is set to 10 dB. The Butterworth filter employs a maximally flat passband response. The passband width was set to 60 (unnormalised frequency) to cover the main lobe of the peak and exclude adjacent frequency components. The passband width is defined in the OPD domain (units: μm) of the FFT amplitude spectrum. The center frequency of the bandpass filter is dynamically determined for each cavity length by identifying the peak corresponding to the target cavity in the FFT amplitude spectrum and taking its OPD value as the center frequency. The lower and upper cutoff frequencies are then calculated as the center frequency ± 30 μm (i.e., half of the bandwidth). Since the center frequency varies with the cavity length, the exact cutoff frequencies are not fixed and are therefore not listed in Table 1.

**Figure 7:**
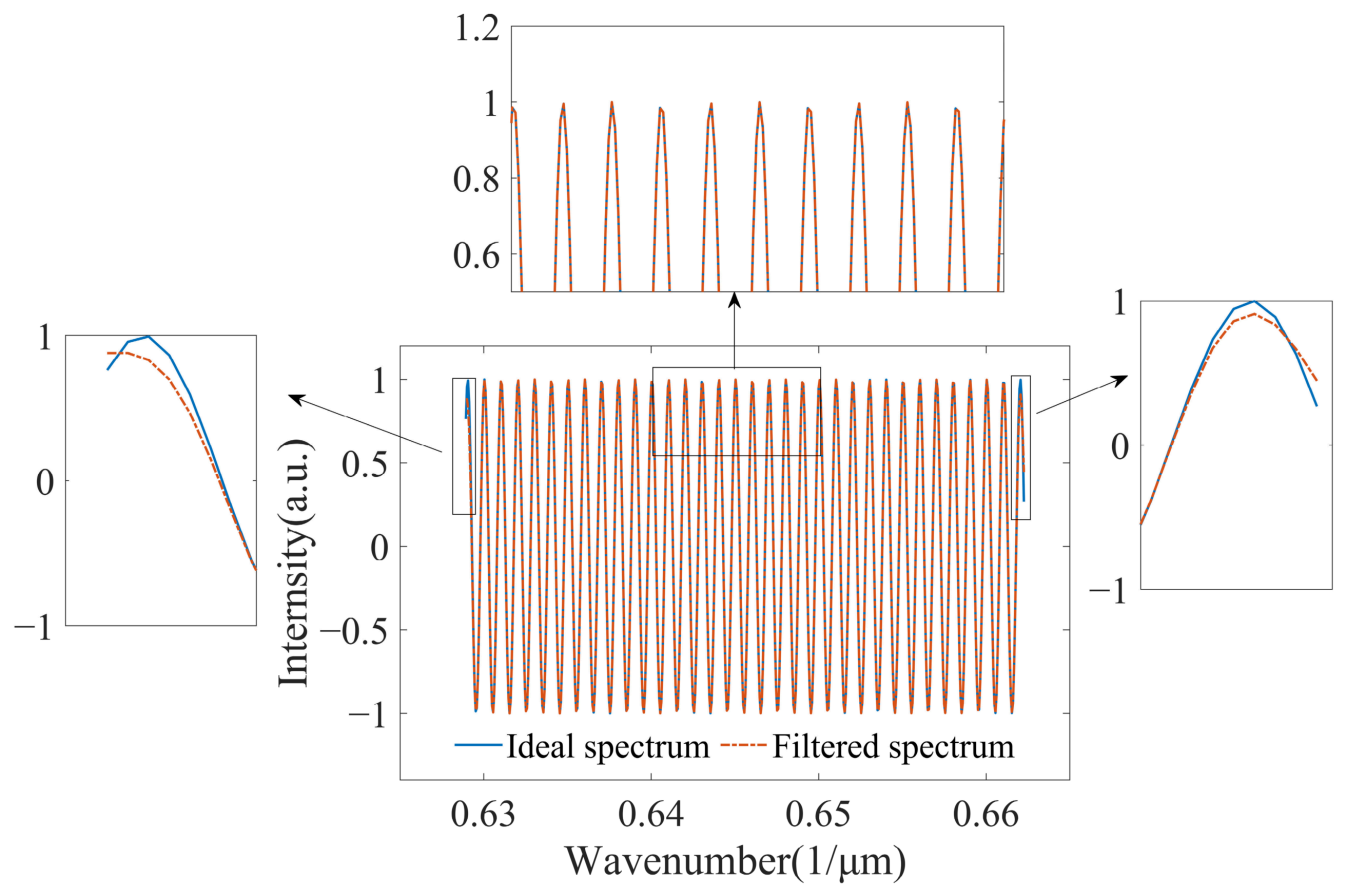
Comparison of the ideal spectrum and the filtered spectrum (Hann window).

The spectral separation performance of six representative FIR window functions—Hann, Hamming, Blackman, Blackman-Harris, Bartlett, and Gaussian—was evaluated. Figure 7 compares the interference spectrum reconstructed using the Hann window with the corresponding ideal spectrum for a cavity length of 500 μm.

The reconstructed spectrum closely matches the overall profile of the ideal spectrum. However, pronounced amplitude distortion appears near both spectral boundaries, whereas the central interference peaks remain well preserved. The remaining five FIR window functions exhibit similar boundary distortion characteristics after filtering. The Hann window is therefore used as a representative example for qualitative analysis, while the subsequent quantitative evaluation includes all six FIR window functions.

**Figure 8:**
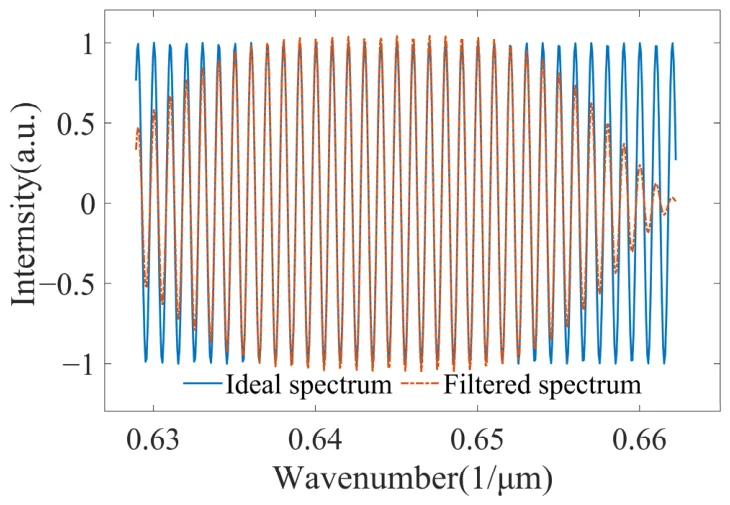
Comparison of the ideal spectrum and the filtered spectrum (Butterworth).

The spectral separation performance of three representative IIR filters—Butterworth, Chebyshev Type I, and Chebyshev Type II—was subsequently evaluated. Figure 8 compares the interference spectrum reconstructed using the Butterworth filter with the corresponding ideal spectrum for a cavity length of 500 μm.

The reconstructed spectrum exhibits a smooth profile with no noticeable boundary distortion, and its overall shape closely matches that of the ideal spectrum. However, slight shifts in the spectral peak positions appear relative to the ideal spectrum. Although these shifts are small, they may introduce errors in subsequent cavity-length demodulation. The other two IIR filters exhibit similar spectral characteristics. Therefore, the Butterworth filter is presented as a representative example, while the subsequent quantitative analysis includes all three IIR filters.

**Figure 9:**
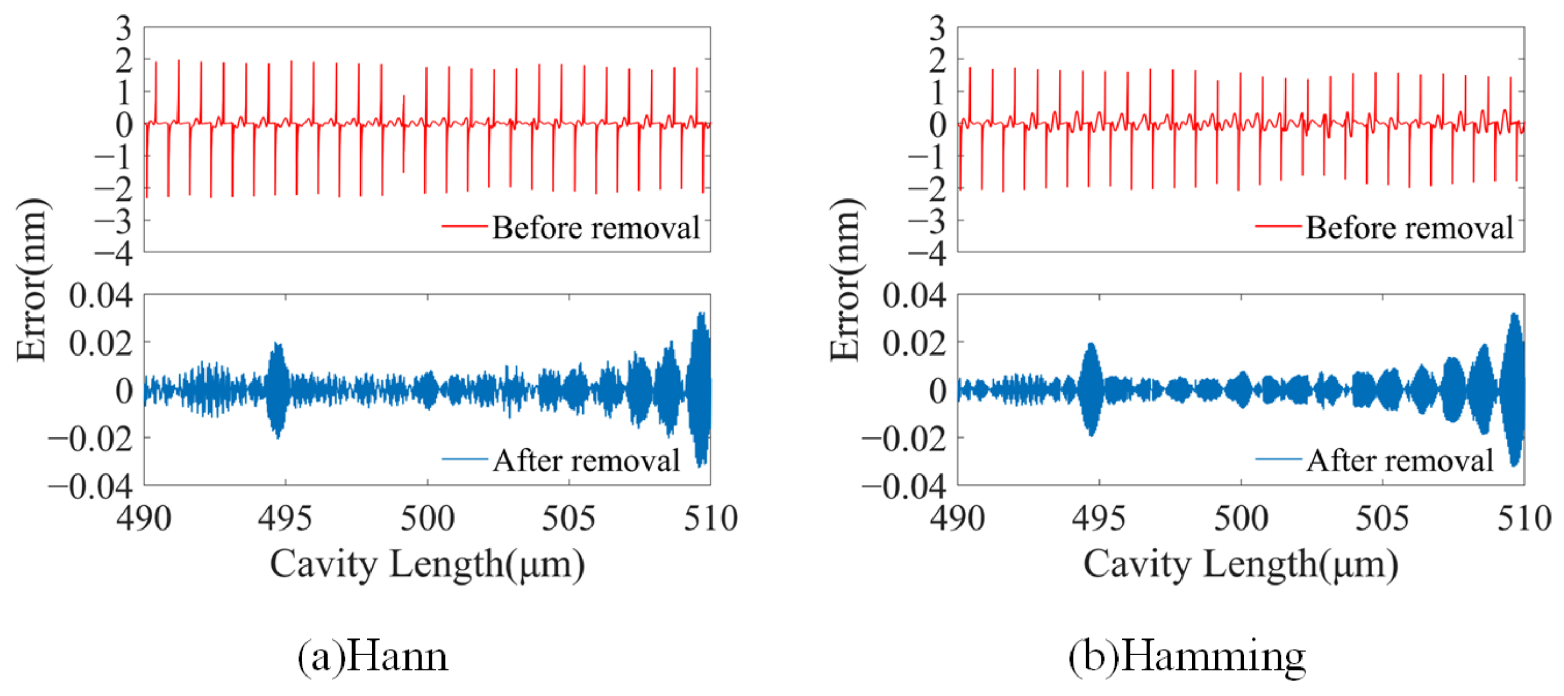

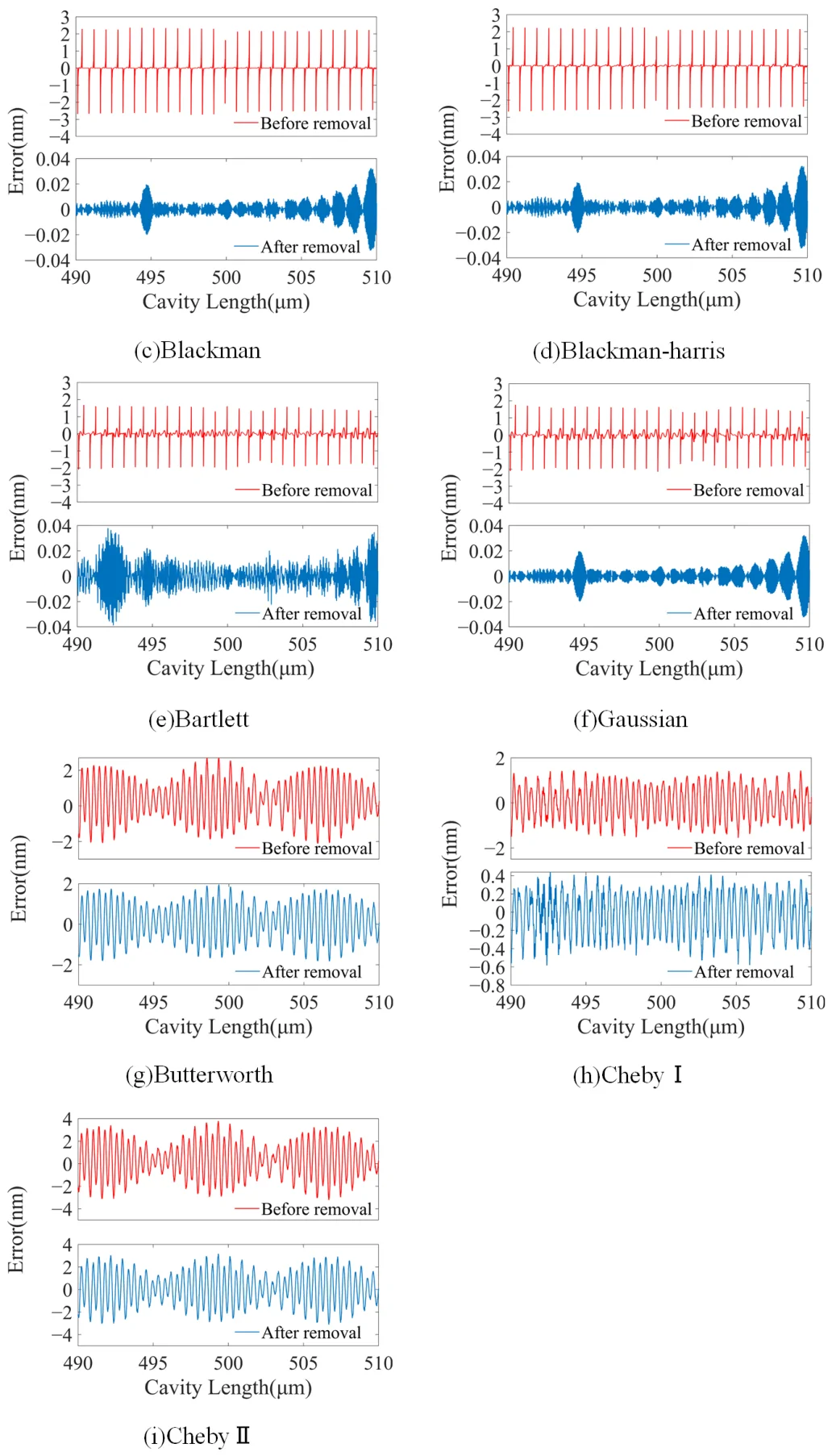
Comparison of demodulation errors with different filters before and after distortion elimination.

Based on the above observations, a distortion-removal strategy was adopted to suppress the influence of filter-induced boundary distortion. Specifically, the distorted interference peaks located at both spectral boundaries were discarded, and only the stable central spectral region was retained for cavity-length demodulation. To implement this strategy quantitatively, the interference peaks located within the first 15% and the last 15% of the entire wavelength range are identified as “boundary-distorted peaks” and are discarded, while only the remaining central 70% of peaks are retained for interference-order fitting and cavity-length calculation. The choice of the 15% threshold was validated by systematically varying the rejection ratio from 5% to 30% in steps of 5% and evaluating the resulting demodulation errors for the six FIR filters. The results show that a rejection ratio of 15% yields consistently low demodulation errors with excellent consistency across all six FIR filters, while deviations from this ratio—either lower (5%) or higher (25% and 30%)—result in larger errors due to insufficient distortion removal or excessive loss of useful information, respectively. This confirms that the method is robust to the exact threshold as long as the distorted edge regions are adequately excluded, and that 15% lies at the center of the stable optimal range. To evaluate the effectiveness of the proposed strategy, nine representative filters were comparatively investigated under identical simulation conditions. For each filter, cavity-length demodulation was performed using two processing schemes: one using all detected interference peaks (before removal) and the other using only the stable central peaks after eliminating the distorted boundary peaks (after removal). The cavity length was varied from 490 to 510 μm with a step size of 1 nm. The demodulation results obtained from the ideal interference spectra without filtering were used as the reference. The simulation results are presented in Figure 9a–f, which compare the cavity-length demodulation errors of the six FIR filters before and after distortion removal, while Figure 9g–i present the corresponding results for the three IIR filters.

In each subfigure, the upper panel presents the demodulation error obtained using all detected interference peaks, while the lower panel shows the corresponding error after rejecting the distorted boundary peaks and retaining only the stable central peaks. The simulation results reveal two distinct characteristics of FIR and IIR filtering.

For the six FIR filters, direct demodulation produced a maximum error of 2.73 nm. After the boundary-distortion rejection strategy was applied, the maximum error decreased to 0.039 nm, comparable to the accuracy achieved under ideal conditions. These results indicate that the demodulation errors introduced by FIR filtering originate primarily from local boundary distortion and can therefore be effectively suppressed by excluding the distorted spectral regions.

In contrast, the three IIR filters exhibit a maximum demodulation error of 3.79 nm before distortion rejection. Even after applying the same processing strategy, the minimum achievable error remains 0.58 nm, substantially higher than that obtained under ideal conditions. This observation indicates that the errors introduced by IIR filtering arise predominantly from global spectral distortion, rather than localized boundary distortion, and therefore cannot be effectively compensated for by rejecting only the boundary peaks.

**Figure 10:**
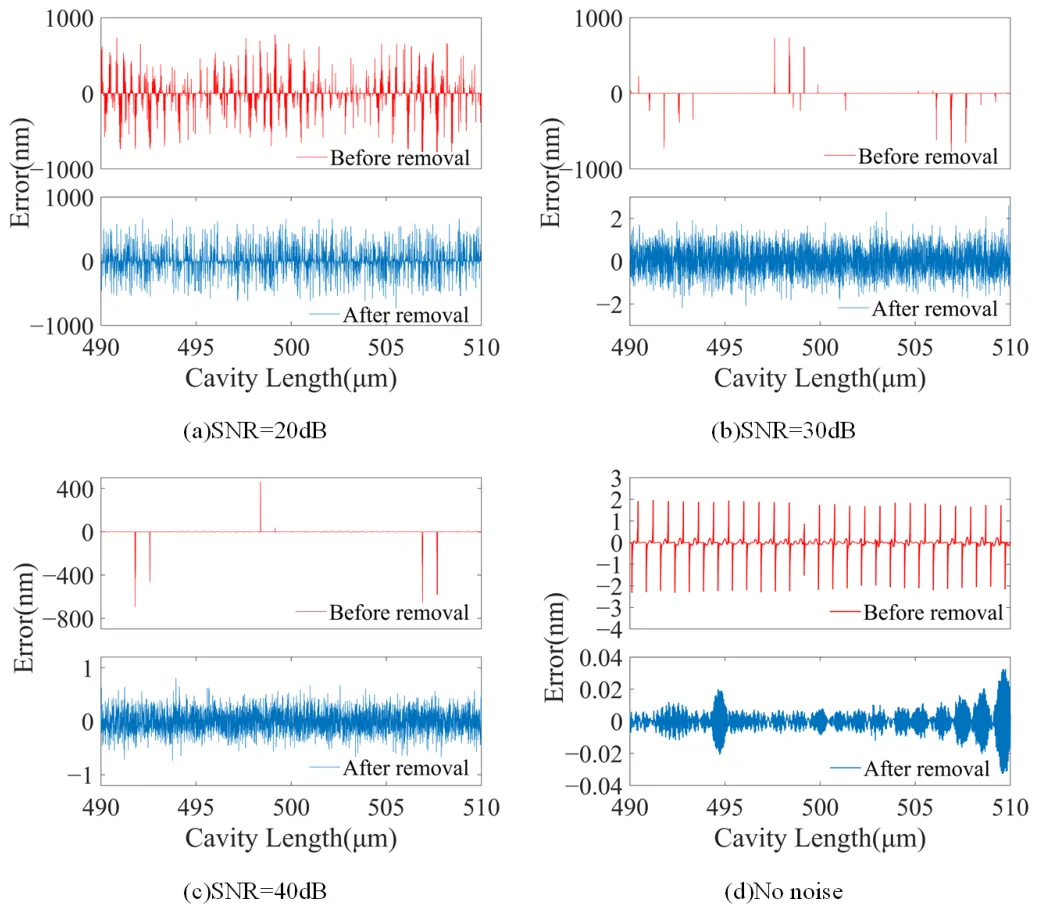
Demodulation error versus SNR for full-spectrum and partial-spectrum demodulation schemes using the Hann-window FIR filter under noisy conditions.

To evaluate the robustness of the proposed method under realistic noise conditions, additional simulations were performed by adding additive white Gaussian noise (AWGN) to the composite interference spectra. Three signal-to-noise ratio (SNR) levels were considered: 20 dB, 30 dB, and 40 dB. For each SNR level, 20 independent noise realizations were generated for each cavity length. The Hann-window FIR filter was selected as a representative example, and both the full-spectrum and partial-spectrum (with 15% boundary rejection) demodulation schemes were applied for comparison. The results are shown in Figure 10.

For SNR ≥ 30 dB, the proposed partial-spectrum scheme maintains high accuracy, with a mean error below 2.60 nm. However, when the SNR decreases to 20 dB, both schemes exhibit occasional demodulation jumps, with errors reaching several hundred nanometers. This is attributed to noise-induced distortion in the filtered spectrum, which leads to misidentification of the interference order and indicates that the signal quality is insufficient to support reliable demodulation. Based on these observations, we conclude that the proposed method maintains reliable performance for SNR ≥ 30 dB, but its effectiveness degrades significantly under lower SNR conditions.

**Figure 11:**
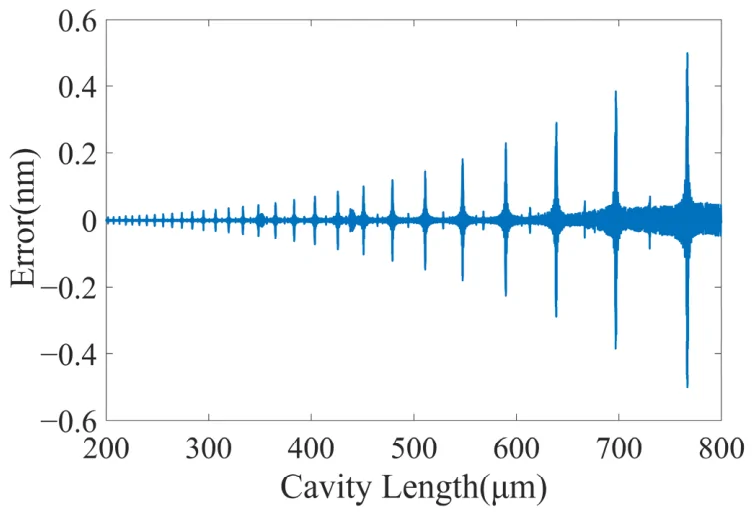
Demodulation error in the 200–800 μm range following FIR filtering combined with distortion rejection.

To further evaluate the robustness of the proposed method, simulations were conducted over a cavity length range of 200–800 μm, as shown in Figure 11.

The results show that when the proposed boundary distortion suppression strategy is combined with FIR filtering, the demodulation error can be kept below 0.53 nm across the entire range, confirming the method’s applicability over a wide range of cavity lengths.

## 4. Experiments

**Figure 12:**
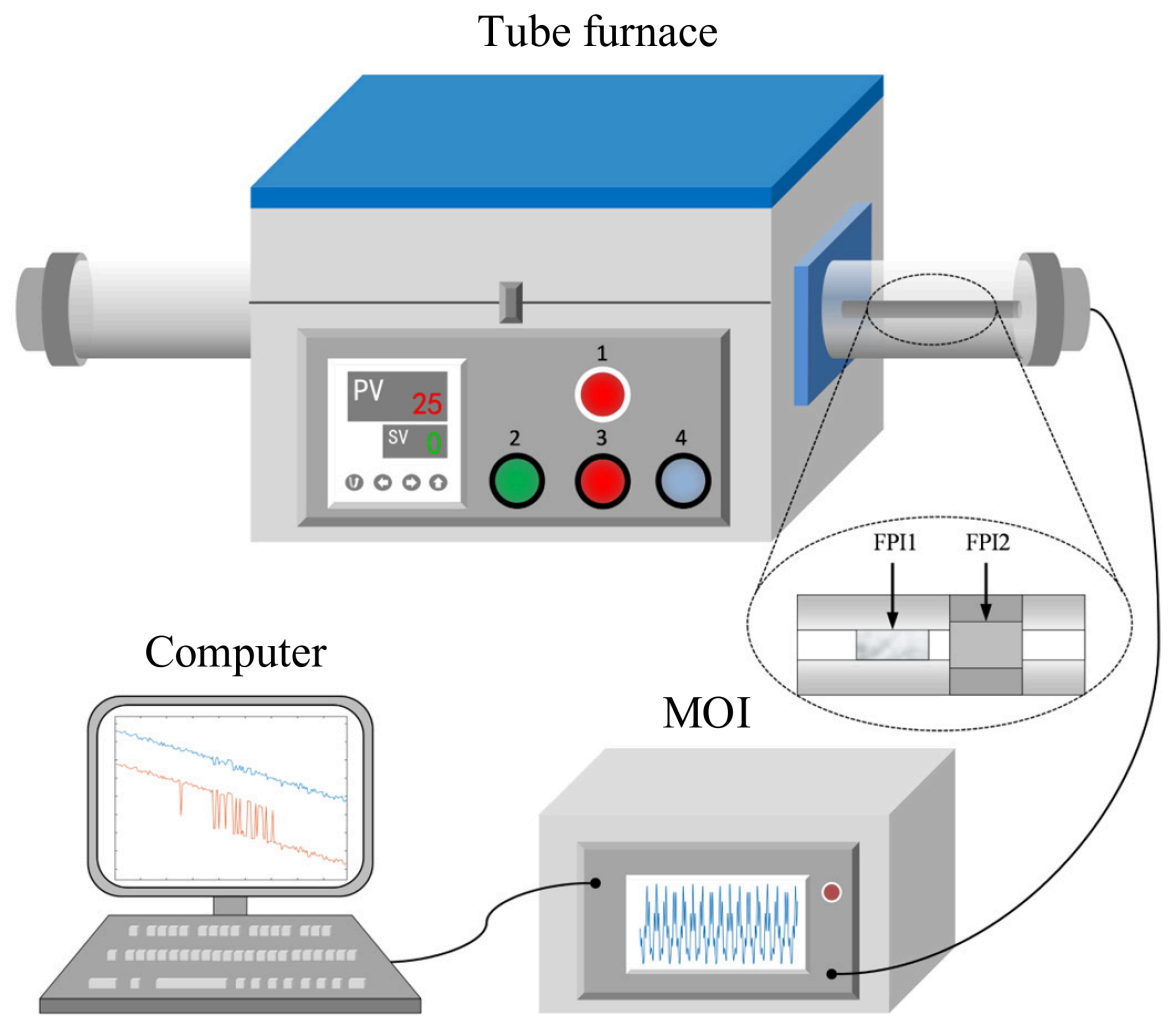
Schematic of the experimental setup.

To experimentally validate the proposed demodulation strategy, a cascaded fiber-optic sensing system consisting of two FP cavities was constructed. A schematic of the experimental setup is shown in Figure 12.

The experimental system consists of a cascaded dual-cavity FP sensor, a tube furnace (OTF-1200X, Hefei Kejing Materials Technology Co., Ltd., Hefei, China), an MOI interrogator (SI155 HS, 1510–1590 nm, LUNA, Roanoke, VA, USA), a platinum resistance thermometer, and a computer. The cascaded dual-cavity FP sensor is formed by connecting two independent intrinsic Fabry–Pérot interferometers (IFPIs). The first FP cavity was fabricated by femtosecond laser direct writing in a polyimide optical fiber, and the second FP cavity was fabricated by femtosecond laser direct writing in an acrylate optical fiber. The initial cavity lengths of *FPI*_1_ and *FPI*_2_ are 997.74 μm and 200.12 μm, respectively. The two sensors were packaged separately and then connected via a flange. The tube furnace provides temperature control with an accuracy of ±0.1 °C. A platinum resistance thermometer with an accuracy of 0.1 °C was inserted into the tube furnace to monitor the real-time temperature, which served as the actual temperature reference. An MOI interrogator with a scan rate of 5 kHz was used to acquire the reflection spectra. The reflection spectra consist of 4000 sampling points over the wavelength range of 1510 nm to 1589.98 nm with a step of 0.02 nm. During the experiment, *FPI*_1_ was positioned inside the tube furnace, while *FPI*_2_ remained outside at room temperature. The reflected interference spectra were continuously acquired by the MOI interrogator and transmitted to the computer for demodulation using the proposed algorithm described in Section 2.

During the cooling process, as the temperature decreased from 74.1 °C to 59.5 °C, the cavity-length demodulation results were continuously recorded. A Hann-window band-pass filter was employed for spectral separation. Two peak-selection strategies were subsequently implemented: (1) full-spectrum demodulation, in which all detected interference peaks were retained; and (2) partial-spectrum demodulation, in which the distorted boundary peaks were discarded, and only the stable central peaks were retained. The two strategies were applied in parallel to the same experimental data, and the corresponding cavity-length variations with temperature were compared.

**Figure 13:**
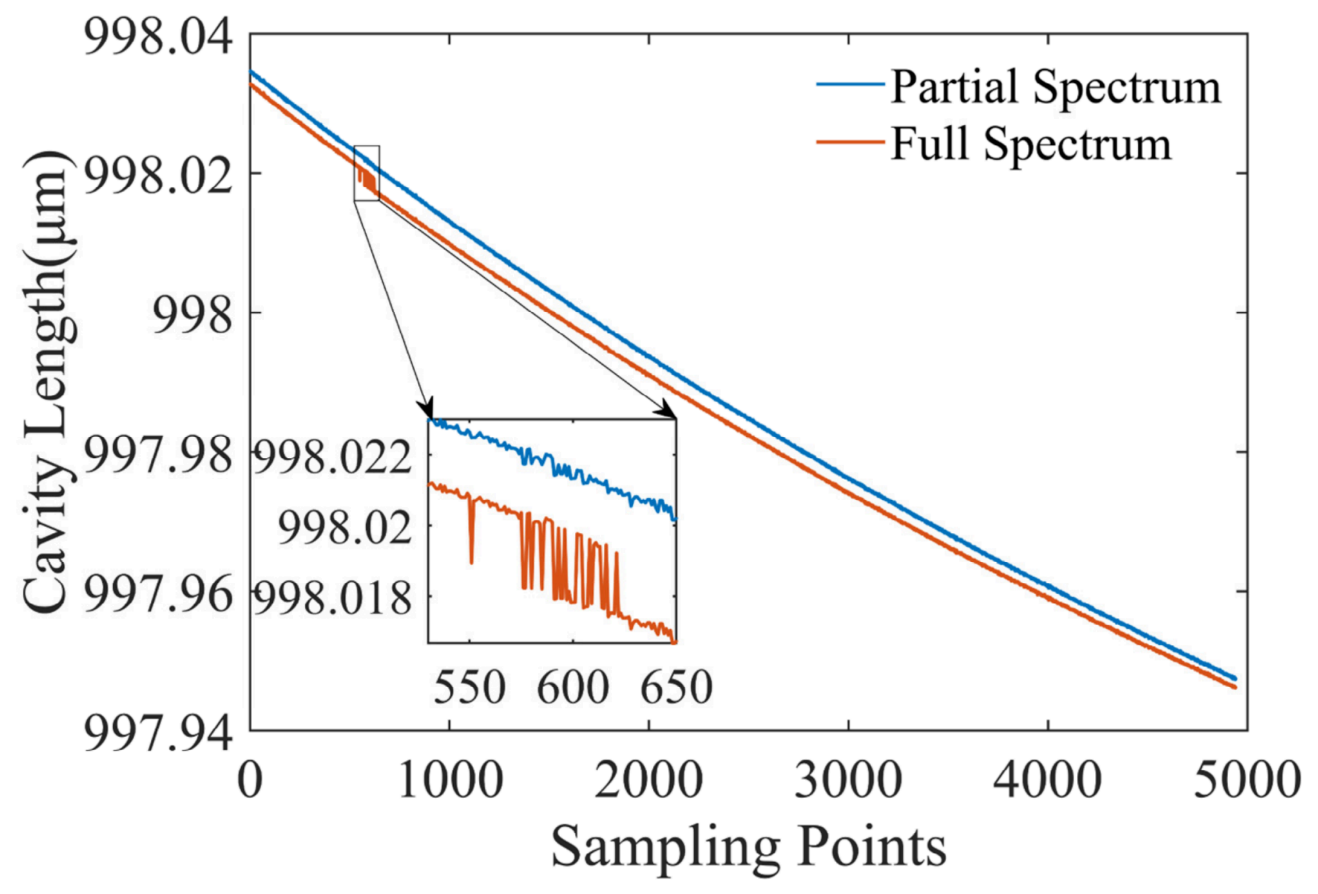
Cavity length variation curves for different peak selection strategies.

Figure 13 compares the cavity-length variations with temperature obtained using the two demodulation strategies. The red and blue curves represent the results of full-spectrum and partial-spectrum demodulation, respectively.

Both demodulation strategies exhibit the same overall temperature-dependent trend, indicating that the proposed boundary-distortion rejection strategy does not affect the underlying sensing response. However, the full-spectrum demodulation results contain several abrupt discontinuities with amplitudes of approximately 2 nm, whereas the partial-spectrum demodulation results remain smooth, continuous, and monotonically decreasing throughout the measurement.

**Figure 14:**
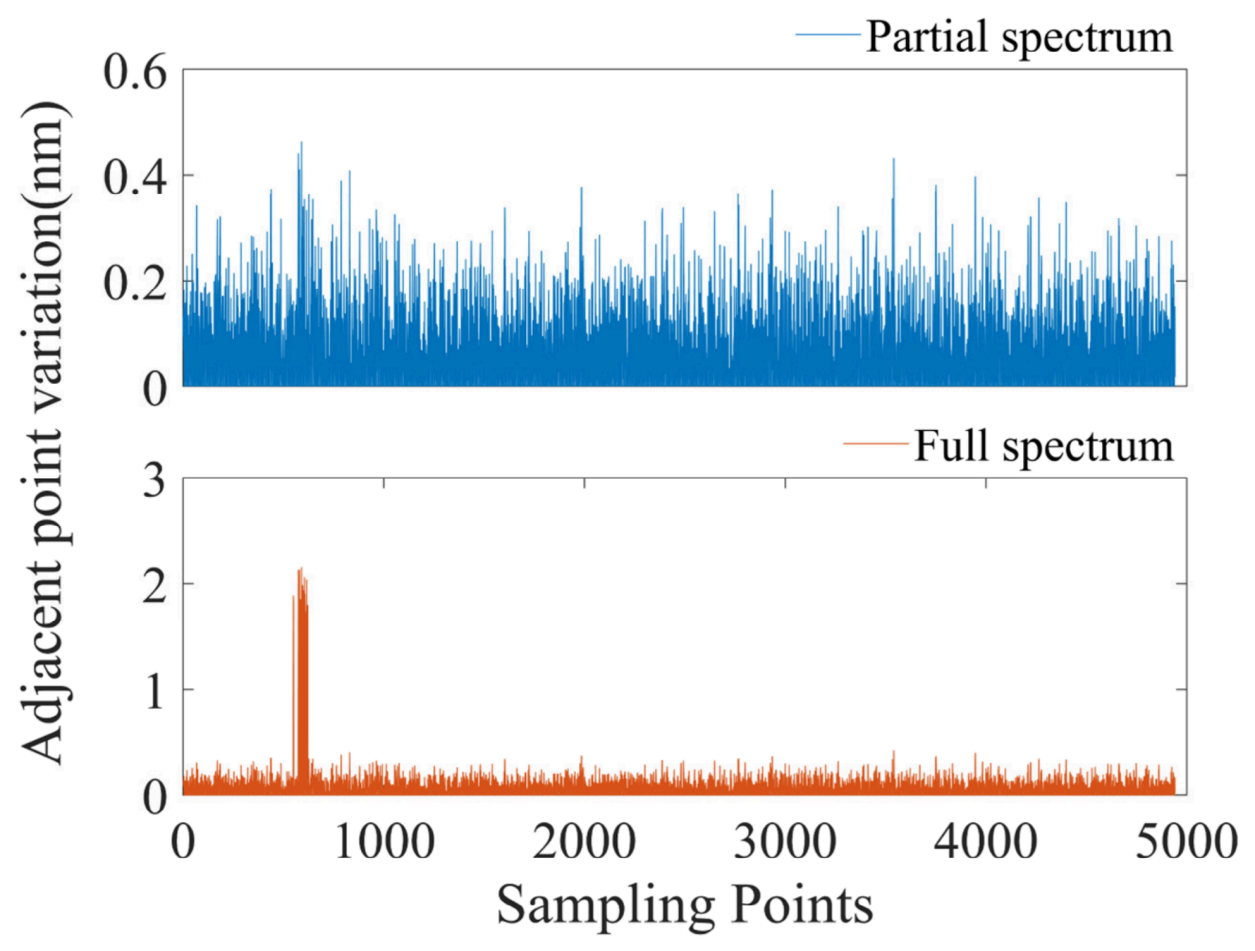
Difference between adjacent points.

**Figure 15:**
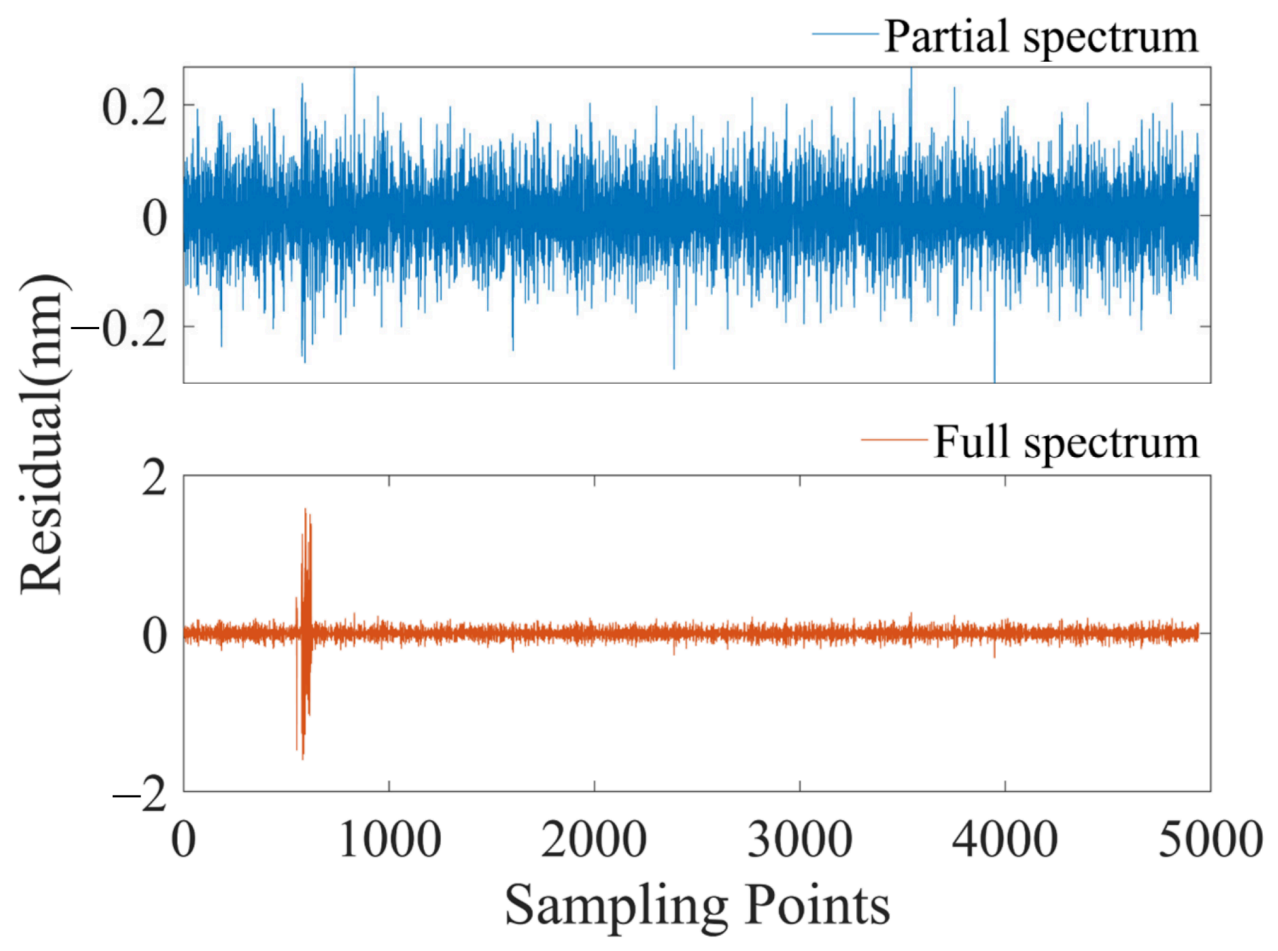
Residual after 5-point moving average detrending.

To quantitatively compare the two demodulation strategies, neighboring-point difference analysis and moving-average residual analysis were performed to evaluate the local continuity and overall stability of the demodulation results. The corresponding results are presented in Figure 14 and Figure 15, respectively.

Neighboring-point difference analysis: The absolute cavity-length differences between adjacent sampling points were calculated to evaluate the local continuity of the demodulation results. The quantitative results are summarized in Table 2. For partial-spectrum demodulation, the maximum adjacent-point difference was 0.464 nm, with a mean of 0.081 nm and a standard deviation of 0.075 nm. In comparison, full-spectrum demodulation yielded a maximum adjacent-point difference of 2.158 nm, a mean of 0.087 nm, and a standard deviation of 0.145 nm. These results demonstrate that partial-spectrum demodulation effectively suppresses abrupt local discontinuities, leading to significantly improved continuity.

Moving-average residual analysis: To remove the global temperature-dependent trend, a five-point moving-average filter was applied to the cavity-length data, and the residual standard deviation was used to quantify the fluctuation level. As summarized in Table 2, the residual standard deviation was 0.072 nm for partial-spectrum demodulation and 0.112 nm for full-spectrum demodulation. The lower residual fluctuation further confirms the superior stability of the proposed boundary-distortion rejection strategy.

Overall, both quantitative analyses consistently demonstrate that rejecting the distorted boundary peaks effectively suppresses abnormal fluctuations and substantially improves the stability and reliability of cavity-length demodulation. These experimental observations agree well with the simulation results presented in Section 3, further validating the proposed boundary-distortion rejection strategy.

## 5. Conclusions

In this paper, we propose a boundary-distortion rejection strategy to improve cavity-length demodulation in fibre-optic multi-FP cavity sensors. Numerical simulations show that FIR filtering mainly introduces local boundary distortion, whereas IIR filtering results in global spectral distortion. Accordingly, the proposed strategy effectively suppresses the demodulation errors caused by FIR filtering, reducing the maximum error from 2.73 nm to below 0.04 nm, but provides only limited improvement for IIR filters. Moreover, simulations over a cavity-length range of 200–800 μm confirm the robustness and broad applicability of the proposed strategy. Experimental results further validate the simulation findings and demonstrate that the proposed method effectively eliminates abnormal demodulation jumps and significantly improves the continuity and stability of cavity-length demodulation. Furthermore, the combination of an FIR filter with the proposed distortion suppression strategy enables high-precision demodulation. It is therefore the preferred choice for applications that require high precision and have ample computational resources. IIR filters offer higher computational efficiency due to their lower order, but their demodulation error is slightly larger than that of the FIR filter scheme. They are therefore more suitable for scenarios where real-time performance takes precedence over precision, or where signal quality is sufficiently high for the effects of distortion to be neglected. In practice, the appropriate filtering scheme should be selected based on specific application requirements. In summary, this paper provides an effective solution for high-precision spectral demodulation in multi-cavity FP sensing systems, and also serves as a reference for filter selection in engineering applications.

## Figures and Tables

**Table 1:** Design parameters of the FIR and IIR band-pass filters.

Filter Type	Window/Type	Order	Taps	Bandwidth	Ripple/Attenuation
FIR	Hann	36	37	60	/
FIR	Hamming	30	31	60	/
FIR	Blackman	40	41	60	/
FIR	Blackman-Harris	46	47	60	/
FIR	Bartlett	32	33	60	/
FIR	Gaussian	32	33	60	α = 2.5
IIR	Butterworth	1	/	60	Maximally flat
IIR	Chebyshev Type I	1	/	60	Rp = 0.1 dB
IIR	Chebyshev Type II	1	/	60	Rs = 10 dB

**Table 2:** Comparative error analysis of two demodulation strategies.

	Partial-Spectrum	Full-Spectrum
Maximum adjacent point difference (nm)	0.464	2.158
Mean adjacent point difference (nm)	0.081	0.087
Standard deviation of adjacent point differences (nm)	0.075	0.145
Standard deviation of moving average residuals (nm)	0.072	0.112

## Data Availability

Data are contained within the article.

## References

[1] Qiang C., Chu C., Wang Y., Yang X., Yang X., Hou Y., Wen X., Teng P., Zhang B., Sivanathan S. (2025). Highly Sensitive Temperature Sensor Based on a UV Glue-Filled Fabry–Perot Interferometer Utilizing the Vernier Effect. Photonics.

[2] Wang H., Hou L., Li J., Yang J. (2025). Fiber-optic axial-strain sensor based on in-cavity micro-bubble fabry-perot interferometer. Opt. Commun..

[3] Hai Z., Su Z., Liang R., Guo M., Zhu H., Chen J., Zhang Q., Chen Y., Lin R., Zhang Y. (2024). Numerical and Experiment Analysis of Sapphire Sandwich-Structure Fabry–Perot Pressure Sensor through Fast Fourier Transform and Mean Square Error Demodulation Algorithm. Materials.

[4] Liu Q., Wang D., Li X., Gao H., Yu D. (2024). Simultaneous measurement of temperature and liquid refractive index based on fiber open Fabry-Pérot cavity and Bragg grating. Optoelectron. Lett..

[5] Wang P., Pan Y., Zhang J., Zhai J., Liu D., Lu P. (2024). Miniaturized and highly sensitive fiber-optic Fabry–Perot sensor for mHz infrasound detection. Photon. Res..

[6] Chen X., Lin Z., Xiong F., Huang H., Chen Y., Tan H., Xu F. (2025). Miniature fiber optic pressure probe for inlet flow field measurements. Sens. Actuators A Phys..

[7] Li L., Fan X., Chen G., Liu Y., Zhang F., Chen Z., Zhang Z., Xu W., Zhang S., Liu Y. (2025). From fish to fiber: 3D-nanoprinted optical neuromast for multi-integrated underwater detection. Nat. Commun..

[8] Gao P., Gong P., Yang Y., Zhou X., Zhang Y., Zhao Y., Warren-Smith S.C., Nguyen L.V., Li X. (2025). Dual-Mode Optical Fiber Sensor with Polydopamine-Functionalized Surface and PDMS-Enhanced Temperature Compensation for Ultrasensitive HCG Detection. Mater. Res. Bull..

[9] Guo M., Zhang Q., Su Z., Zhu H., Liang R., Zhang C., Wang B., Guo B., Zheng Y., Chen J. (2024). Simultaneous measurement of gas pressure and temperature sensor based on FP interference using hollow core Bragg fiber. IEEE Sens. J..

[10] Wang Z., Chen J., Wei H., Liu H., Ma Z., Chen N., Chen Z., Wang T., Pang F. (2020). Sapphire Fabry–Perot interferometer for high-temperature pressure sensing. Appl. Opt..

[11] Qi X., Bing M., Zhi T., Ruo L., Zhi M. (2024). Dynamic demodulation technique for tandem dual-cavity EFPI sensors enabling simultaneous temperature measurement in harsh environment. IEEE Sens. J..

[12] Deng H., Jiang Y., Zhang Y., Xie S. (2024). A high-accuracy demodulation algorithm based on the frequency shift technique for interrogating fiber-optic dual-cavity Fabry–Perot sensors. IEEE Sens. J..

[13] Ren Q., Jia P., Qian J., Wang J., Liu W., Xiong J. (2022). Three-wavelength Dynamic Demodulation Technique for Multi-cavity Fiber Fabry-Perot Sensor. Acta Photonica Sin..

[14] Li Z., Wang S., Jiang J., Sang M., Yu Y., Liu W., Zhang P., Liu T. (2021). Real-time pressure measurement method based on rapid phase demodulation of multi-cavities FP sensor. IEEE Sens. J..

